# Co-infection VIH-tuberculose pulmonaire aux cliniques universitaires de Lubumbashi: aspects épidémiologiques et cliniques

**DOI:** 10.11604/pamj.2025.51.65.43951

**Published:** 2025-07-04

**Authors:** Patrick Mutombo Tshibang, Georges Yumba Numbi, Eugénie Tshiami Bukasa, Evariste Yav Tshibind, Thierry Kisoka Kimbungu, Serge Ngoie Muleka

**Affiliations:** 1 Département de Médecine Interne, Faculté de Médecine, Université de Lubumbashi, Lubumbashi, République Démocratique du Congo

**Keywords:** VIH, tuberculose pulmonaire, Lubumbashi, HIV, pulmonary tuberculosis, Lubumbashi

## Abstract

**Introduction:**

l'objectif de cette étude est de présenter un aperçu des caractéristiques épidémiologiques et cliniques de la co-infection VIH-tuberculose pulmonaire aux cliniques universitaires de Lubumbashi.

**Méthodes:**

il s’agit d’une étude descriptive transversale qui a porté sur l’analyse des dossiers de patients tuberculeux qui ont été hospitalisés au service de médecine interne de cliniques universitaires de Lubumbashi, dans le sud-est de la République démocratique du Congo, au cours de l’année 2019.

**Résultats:**

la co-infection VIH-tuberculose a concerné 37 patients sur un total de 66 ayant été colligés (56,0%). La tuberculose du patient infecté par le VIH en comparaison avec celle du patient non infecté, dans notre série, était caractérisée par une relative prédominance des patients d´âge supérieur ou égale à 40 ans (56,8% vs 34,4%, p=0,004), du sexe féminin (78,9% vs 46,8%, p=0,01), de cas de récidive (37,8% vs 27,5%), et de l´antécédent de diabète sucré (8,1% vs 3,4%). Un peu plus du tiers des coinfectés, soit 37,4%, provenaient des quartiers périphériques de la ville de Lubumbashi, caractérisés pour la plupart d’entre eux par une conjoncture socio-économique défavorable. Les formes à microscopie négative, donc sans confirmation bactériologique, étaient les mieux représentées parmi les co-infectés (48,6% vs 34,4%). Le taux de décès à l´issue de l´hospitalisation a été de 13,5% chez les co-infectés. Il était associé aux facteurs ci-après: la tuberculose à microscopie positive (21,0% vs 5,5%), le sexe masculin (18,1% vs 6,6%), le diabète sucré (33,3% vs 11,7%) et l´âge supérieur à 40 ans (19,0% vs 6,2%).

**Conclusion:**

la co-infection VIH-tuberculose pulmonaire est donc une situation fréquente en hospitalisation aux cliniques universitaires de Lubumbashi avec un profil épidémiologique et clinique marqué par la présence de facteurs pouvant impacter négativement son pronostic à court terme.

## Abstract (en)

**Introduction:**

this study aims to provide an overview of the epidemiological and clinical characteristics of HIV–pulmonary tuberculosis (TB) co-infection among patients treated at the University Clinics of Lubumbashi.

**Methods:**

we conducted a descriptive cross-sectional study based on the analysis of medical records of patients with tuberculosis admitted to the Internal Medicine Department of the University Clinics of Lubumbashi, located in the southeastern region of the Democratic Republic of the Congo, during the year 2019.

**Results:**

HIV–tuberculosis co-infection was identified in 37 out of 66 medical records reviewed (56.0%). Compared to HIV-negative patients, TB in HIV-positive individuals was more frequently observed in patients aged 40 years and older (56.8% vs. 34.4%, p = 0.004), in females (78.9% vs. 46.8%, p = 0.01), in cases of recurrent TB (37.8% vs. 27.5%), and in those with a history of diabetes mellitus (8.1% vs. 3.4%). Just over one-third of co-infected patients (37.4%) originated from the peripheral neighborhoods of the city of Lubumbashi, most of which are characterized by unfavorable socioeconomic conditions. Smear-negative forms of tuberculosis, lacking bacteriological confirmation, were predominant among co-infected patients (48.6% vs. 34.4%). Mortality rate at discharge was 13.5% among the co-infected patients. Mortality was associated with the following factors: smear-positive TB (21.0% vs. 5.5%), male sex (18.1% vs. 6.6%), diabetes mellitus (33.3% vs. 11.7%), and age over 40 years (19.0% vs. 6.2%).

**Conclusion:**

HIV–pulmonary tuberculosis co-infection is a frequent condition among inpatients at the University Clinics of Lubumbashi. The epidemiological and clinical profile is marked by the presence of factors that may adversely affect short-term prognosis.

## Introduction

La tuberculose pulmonaire est un problème majeur de santé publique dans le monde, en particulier dans les pays en développement ou à faible revenu où elle est responsable d'une grande mortalité et morbidité [1,2]. Elle constitue en effet la principale affection opportuniste de l'infection à VIH, réalisant un duo mortel, dans la mesure où ces deux pathologies s'accélèrent mutuellement dans leur évolution avec, in fine, une implication pronostique négative sur l'état de santé du patient [1,3].

L’Organisation mondiale de la Santé (OMS) a rapporté que près de 9,9 millions de personnes ont contracté la tuberculose en 2020, parmi elles 1,5 million de décès dont 214000 étaient concernés par la co-infection VIH-tuberculose; plus de 95% de ces décès ou des cas sont survenus dans les pays en développement [1]. En Afrique subsaharienne, la prévalence de la co-infection VIH-tuberculose, estimée en 2019 par une méta-analyse à 31,8%, reste néanmoins élevée en dépit d’une amélioration positive constatée dans certains pays, se traduisant par une diminution de l’incidence et du taux de mortalité associée à la tuberculose [4]. L’infection à VIH demeure dans cette région du monde une barrière pour une prévention et un contrôle efficace de la tuberculose [4-6].

La République démocratique du Congo fait partie encore de ces 22 pays qui supportent près de 80% du poids de la tuberculose dans le monde. Elle figure également parmi les pays qui portent le fardeau de la co-infection VIH-tuberculose [7-9]. Eu égard à ce qui précède, la tuberculose pulmonaire devrait être un motif majeur d´hospitalisation dans l´infection à VIH. En dépit du rôle qu´elle joue dans la morbidité et mortalité hospitalière, la co-infection VIH-tuberculose pulmonaire souffre encore du nombre relativement insuffisant des études, notamment celles rapportant les caractéristiques de son profil épidémiologique et clinique en milieu hospitalier de niveau tertiaire comme les cliniques universitaires de Lubumbashi. Ainsi, l´objectif poursuivi par cette étude est celui de présenter une version hospitalière des caractéristiques épidémiologiques et cliniques associées à la co-infection VIH-tuberculose pulmonaire au service de médecine interne des cliniques universitaires de Lubumbashi.

## Méthodes

**Type et cadre d´étude:** la présente étude est descriptive, transversale. Elle a été menée du 1^er^ janvier au 31 décembre 2019 au service de médecine interne des cliniques universitaires de Lubumbashi, centre Hospitalier général de référence située dans le sud-est de la république démocratique du Congo.

**Critères d´inclusion:** ont été inclus les patients tuberculeux avec sérologie VIH connue, qui ont été hospitalisés dans le service de médecine interne durant la période d´étude.

**Variables d´études:** les variables sur lesquelles a reposé l´étude ont été catégorisées en 2 groupes: les variables sociodémographiques: l´âge, le sexe, la commune de résidence par rapport à la ville de Lubumbashi; les variables cliniques: le statut sérologique VIH, les principaux antécédents pathologiques médicaux, les signes cliniques, la forme clinique et l´issue en hospitalisation. Concernant la variable âge, la population d´étude a été divisée en 3 groupes: les patients de moins de 40 ans, ceux avec un âge compris entre 40 et 60 ans et ceux de plus de 60 ans. Les antécédents médicaux systématiquement recherchés chez les patients ont été: la tuberculose antérieure, la notion de contage, le diabète sucré, la consommation régulière de l´alcool et du tabac. Par rapport à la forme clinique, la tuberculose pulmonaire était catégorisée en deux types: la tuberculose pulmonaire bactériologiquement confirmée, c´est-à-dire avec coloration de Ziehl positive dans le crachat (TPM+) et la tuberculose pulmonaire cliniquement diagnostiquée, en présence d´un examen négatif de crachat dans un contexte d´un tableau clinique ou radiologique évocateur (TPM-). La co-infection VIH-tuberculose (TBVIH+) impliquait la présence d´un statut VIH positif soit rapporté déjà lors l´anamnèse à l´admission ou diagnostiqué lors du bilan biologique initial du patient en se basant sur l´algorithme décisionnel édicté par le programme national de lutte contre l´infection à VIH en République démocratique du Congo. L´issue de l´hospitalisation était une variable binaire qui impliquait le décès ou non du patient.

**Collection de données:** les données ont été récoltées, au moyen d'une fiche d'enquête, dans les dossiers médicaux de patients structurés comme suit: l'anamnèse, l'examen clinique, les diagnostics, la prise en charge ainsi que l'évolution. Elles étaient en rapport avec variables d'étude et ont été ensuite encodées sur une feuille Excel 2016.

**Analyse statistique:** le logiciel Epi Info 7 a servi pour l’analyse tant univariée que bivariée des données encodées. Pour les variables quantitatives, le calcul de la moyenne a été utilisé. En revanche, pour les variables qualitatives, l’analyse a tourné autour du calcul de proportions ou de fréquences relatives. La comparaison de moyenne a fait recours au test t et celle de proportion au test de khi- carré ou de Fisher exact. Une valeur de p inférieure à 0,05 a impliqué une différence statistiquement significative.

**Considérations éthiques:** cette étude a été menée dans le respect des normes éthiques en vigueur. Elle a obtenu l’approbation du Comité d’Éthique de la Faculté de Médecine de l’Université de Lubumbashi (nº UNILU/CEM/034/2018).

## Résultats

La présente étude a colligé un total de 66 patients tuberculeux, parmi lesquels 37 présentaient une co-infection VIH-tuberculose, soit une prévalence de 56,0%.

**Données sociodémographiques:** la co-infection VIH-tuberculose pulmonaire a été caractérisée dans un peu plus de la smoitié des cas (56,8%) par un âge compris entre 40 et 60 ans. Une association statistiquement a été retrouvée entre le sexe féminin et la co-infection VIH-tuberculose pulmonaire (78,9% vs 46,8%, p=0,01). La majorité des cas de co-ïnfection, par ailleurs, résidait dans la commune annexe de la ville de Lubumbashi (37,4%) suivie de la commune de Lubumbashi (24,3%), et celle de Kampemba (16,2%). Les communes de Katuba et de Kamalondo étaient les moins représentées avec respectivement 2,7% et 0,0% (Tableau 1).

**Tableau 1:** caractéristiques sociodémographiques

Caractéristiques sociodémographiques	Statut sérologique VIH		P-value
	VIH+(n=37)	VIH-(n=29)	
Age moyen	38,6±10,5 ans	36,7±14,8 ans	0,5
**Tranche d’âge(an)**			
<40	16(43,2%)	19(65,5%)	0,004
40-60	21(56,8%)	7(24,1%)	
>60ans	0(0,0%)	3(10,3%)	
**Sexe**			
Sexe féminin (n=19)	15(78,9%)	4(21,1%)	0,01
Sexe masculin (n=47)	22(46,8%)	25(53,2%)	
**Commune de résidence**			
Annexe	14(37,4%)	7(24,5%)	0,04
Kampemba	6(16,2%)	6(20,6%)	
Katuba	1(2,7%)	4(13,7%)	
Kenya	2(5,4%)	0(0,0%)	
Lubumbashi	9(24,3%)	10(34,4%)	
Rwashi	5(13,5%)	0(0,0%)	
Kamalondo	0(0,0%)	2(6,9%)	

Virus de l'immunodéficience Humaine (VIH)+: sérologie VIH+; VIH-: sérologie VIH-

**Données cliniques:** concernant les principaux antécédents médicaux, une prédominance relative en termes de fréquence a été constatée pour la notion tuberculose antérieure (37,8%) et de diabète sucré (8,1%). En revanche la consommation d’alcool (42,2%), le tabagisme (24,1%) ainsi que la notion de contage (6,9%) ont été les antécédents les plus fréquents chez les patients tuberculeux non infectés par le VIH. Le tableau clinique de la tuberculose chez le patient infecté par le VIH était dominé par la toux (97,3%), la douleur thoracique (81,0%), l´asthénie (89,1%) et l´anorexie soit (75,6%).

La co-infection VIH-tuberculose pulmonaire a été caractérisée dans notre série sur le plan diagnostique par une prédominance relative de formes à microscopie négative (TPM-), donc cliniquement diagnostiquées soit 48,6% contre 34,4%. Les formes bactériologiquement confirmées (TPM+) ont, quant à elles, caractérisé la tuberculose du patient non infecté par le VIH. L’issue de l’hospitalisation a été marquée par un taux de décès dans la co-infection VIH-tuberculose pulmonaire de 13,5%. Ce taux de décès était relativement élevé chez les patients de sexe masculin (18,1%), chez ceux âgés de plus de 40 ans (19,0%), chez ceux ayant présenté une forme bactériologiquement confirmée (21,0%) ou chez ceux atteints du diabète sucré (33,3%) (Tableau 2 et Tableau 3).

**Tableau 2:** caractéristiques cliniques

Caractéristiques cliniques	Statut sérologique VIH		
	VIH+(n=37)	VIH-(n=29)	P-value
**Antécédents médicaux**			
Consommation d’alcool (n=25)	11(29,7%)	14(42,2%)	0,12
Tabac (n=16)	9(24,2%)	7(24,1%)	0,98
Tuberculose antérieure (n=22)	14(37,8%)	8(27,5%)	0,38
Diabète sucré (n=4)	3(8,1%)	1(3,4%)	0,43
Notion de contage (n=4)	2(5,5%)	2(6,9%)	1,0
**Signes cliniques**			
Amaigrissement	29(78,3%)	25(86,2%)	0,41
Anorexie	28(75,6%)	21(72,4%)	0,76
Asthénie	33(89,1%)	23(79,3%)	0,26
Douleur thoracique	30(81,0%)	18(62,0%)	0,08
Fièvre	30(81,0%)	25(86,2)	0,57
Hémoptysie	7(18,9%)	8(27,5%)	0,40
Toux	36(97,3%)	26(89,6%)	0,31
Transpiration nocturne	24(64,8%)	19(65,5%)	0,95
**Formes cliniques**			
TPM+ (38)	19(51,4%)	19(65,6%)	0,24
TPM- (28)	18(48,6%)	10(34,4%)	
**Issue de l’hospitalisation**			
Décès oui (10)	5(13,5%)	5(17,2%)	0,67
Décès non (56)	32(86,5%)	24(82,8%)	

TPM+: tuberculose pulmonaire à microscopie positive ou bactériologique confirmée; TPM-: tuberculose pulmonaire à microscopie négative ou cliniquement diagnostiquée; Virus de l'immunodéficience humaine (VIH)+: sérologie VIH+; VIH-: sérologie VIH-

**Tableau 3:** issue de l’hospitalisation de patients co-infectés en fonction de quelques caractéristiques sociodémographiques et cliniques

Caractéristiques sociodémographiques et cliniques	Issue de l’hospitalisation		P-value
	Décès oui (n=5)	Décès non (n=32)	
**Tranche d’âge**			
<40 ans (n=16)	1(6,2%)	15(93.8%)	0,36
40-60 (n=21)	4(19,0%)	17(81%)	
>60 ans (n=0)	0(0,0%)	0(0,0%)	
**Sexe**			
Sexe masculin (n=22)	4(18,1%)	18(81,9%)	0,31
Sexe féminin (n=15)	1(6,6%)	14(93,4%)	
**Formes cliniques**			
TPM+ (n=19)	4(21,0%)	15(79,0%)	0,33
TPM (n=18)	1(5,5%)	17(94,4%)	
**Diabète sucré**			
Oui (n=3)	1(33,3%)	2(66,7%)	1,0
Non (n=34)	4(11,7%)	30(88,3%)	

## Discussion

La prévalence de la co-infection VIH-tuberculose pulmonaire a été de 56% dans notre série. Elle est globalement comparable à celle trouvée dans bon nombre d’études menées sur le continent africain en général [4,5,10-13] et en particulier en République démocratique du Congo [7,8]. Cette fréquence relativement élevée rappelle une fois de plus le rôle joué par l’infection à VIH, en l’occurrence non contrôlée ou méconnue, dans la survenue de formes graves de tuberculose [11], lesquelles peuvent être isolées ou associées à d’autres maladies opportunistes. Ces formes graves peuvent mettre en jeu le pronostic vital à court terme et ont indiqué d’emblée une prise en charge urgente en hospitalisation au service de médecine interne des cliniques universitaires.

La co-infection VIH pulmonaire est relativement fréquente chez les femmes comparativement aux hommes (78,9% vs 46,8%, p=0,01). Plusieurs auteurs en Afrique ont trouvé un résultat similaire dans leur série avec des proportions de femmes pouvant dépasser 50% chez les coinfectés [6-8,10]. Cette prédominance féminine trouve explication dans l'influence qu'a l'infection à VIH sur la diminution du ratio homme/femme des cas de tuberculose débouchant sur une tendance nette à la féminisation de la coïnfection VIH-tuberculose [7,8,10].

Les cas de co-infection VIH-tuberculose pulmonaire se sont recrutés dans la majorité de cas chez les patients dont l´âge était compris entre 40 et 60 ans (56,8%) et ce contrairement aux séronégatifs chez qui ont prédominé les moins de 40 ans (65,5%). Akilimali *et al*. Ainsi que Wa *et al*. ont fait le même constat dans leurs travaux [7,8]. Ce résultat suggère que l'infection à VIH augmenterait l'âge de survenue de la tuberculose, maladie touchant en principe dans la majorité des cas les moins de 40 ans [11,14].

Près du tiers des coinfectés résidaient dans la commune annexe, soit 37,4% suivi de celle de Lubumbashi soit 24,3%. Le transfert de cas de co-infection VIH-tuberculose pulmonaire des centres hospitaliers de bas échelons situés notamment dans la commune annexe vers les cliniques universitaires de Lubumbashi devrait en partie expliquer nos résultats. En outre, ces dernières sont situées dans la commune de Lubumbashi, laquelle est l'épicentre des activités économiques, politiques, sociales et sanitaires de la ville de Lubumbashi, conférant à cette entité territoriale le statut de site de convergence des autres communes [8,14].

Les antécédents médicaux relativement fréquents chez les tuberculeux co-infectés comparativement aux non infectés ont été la notion d’une tuberculose antérieure (37,8% vs 27,5%) ainsi que celle de diabète sucré (8,1% vs 3,4%). La baisse de l'immunité liée à l'infection à VIH augmente le risque de récidive de la tuberculose le plus souvent par réinfection que par réactivation endogène dans les zones à forte incidence [3,15]. Une méta-analyse a trouvé, en Afrique subsaharienne, une prévalence du diabète sucré dans la co-infection VIH-tuberculose de 8,9%, laquelle est légèrement supérieure à celle des patients tuberculeux non infectés, mettant ainsi en exergue le rôle possible de l’infection à VIH, ainsi que celui des effets secondaires du traitement antirétroviral, dans la survenue du diabète sucré [16]. La clinique de la tuberculose pulmonaire chez les co-infectés est restée dominante dans notre série par la toux, la douleur thoracique, l’asthénie et l’anorexie. Une différence significative n’a pas été constatée entre les patients infectés par VIH et les non infectés quant à l’expression clinique de la tuberculose pulmonaire dans notre série. Akilimali *et al*. ont fait le même constat à Kinshasa [7].

En rapport avec le diagnostic, la co-infection VIH-tuberculose pulmonaire a été caractérisée par une prédominance relative des formes à microscopie négative, donc sans confirmation bactériologique (48,6% vs 34,4%). Plusieurs auteurs ont trouvé également un résultat similaire [6-8,10,12]. En effet, l’infection à VIH diminue la sensibilité de l’examen direct de crachat par la coloration de Ziehl et favorise l’expression de formes pulmonaires pauci bacillaires et extra pulmonaires [10]. L'intérêt de réaliser ou de généraliser les tests relativement plus sensibles comme le GeneXpert s'impose pour améliorer la fiabilité diagnostique.

À l’issue de l’hospitalisation, le taux de décès associé à la co-infection VIH-tuberculose pulmonaire était de 13,5%, non différent de celui des patients non co-infectés. Ce taux de décès était relativement élevé chez les patients chez qui les caractéristiques suivantes ont été retrouvés: l'âge supérieur à 40 ans, le sexe masculin, le diabète sucré ainsi que la forme clinique bactériologiquement confirmée (TPM). Wa *et al*. à Lubumbashi ont trouvé un taux de décès de 8,8% chez les coinfectés avec presque les mêmes facteurs associés que les nôtres [8].

Les patients de plus de 40 ans pourraient, en dehors de l’infection à VIH, avoir d’autres comorbidités, lesquelles leur prédisposeraient à un taux de décès relativement élevé [7,8]. Le sexe masculin serait peut-être l'objet des consultations tardives et aurait ainsi un risque élevé de présenter des formes sévères ou compliquées. Le diabète sucré en l'occurrence non contrôlé ou compliqué, étant connu comme un facteur de mauvais pronostic dans la tuberculose [17], devrait être considéré comme un facteur additif de mortalité dans la co-infection VIH-tuberculose pulmonaire. Les formes bactériologiquement confirmées ont habituellement un taux de décès et une contagiosité élevée, leur accordant un caractère prioritaire sur le plan thérapeutique [13,14].

## Conclusion

En milieu hospitalier où la présente étude a été menée, la co-infection VIH-tuberculose pulmonaire est mieux représentée en termes de fréquence. Elle est caractérisée par la prédominance des formes à microscopie négative et a touché avec prédilection les patients à partir de la quarantaine d'âge, parmi lesquels les femmes ont représenté une part considérable. La notion de tuberculose antérieure ou de diabète sucré a été rapportée parmi les antécédents médicaux les plus fréquents. De ce qui précède, la co-infection VIH-tuberculose a un profil épidémiologique et clinique marqué par des facteurs pouvant impacter négativement son pronostic à court terme.

### Etat des connaissances sur le sujet

- La co-infection VIH-tuberculose est un problème majeur de santé publique en république démocratique du Congo;
- Le duo VIH-tuberculose est responsable d’une grande morbi mortalité.

### Contribution de notre étude à la connaissance

- La co-infection VIH-tuberculose pulmonaire est une situation fréquente en milieu hospitalier de niveau tertiaire, en particulier aux cliniques universitaires de Lubumbashi;
- La présence de facteurs à caractère péjoratif, parmi lesquelles l´âge avancé ou les comorbidités telles que le diabète sucré ou les infections opportunistes liées au VIH, devrait avoir un impact négatif sur le pronostic à court terme;
- La présente étude rappelle encore une fois l'intérêt de redoubler les efforts pour davantage améliorer la prise en charge tant curative que préventive de ces deux pandémies.
